# Shape-controlled synthesis of zinc nanostructures mediating macromolecules for biomedical applications

**DOI:** 10.1186/s40824-022-00252-y

**Published:** 2022-02-02

**Authors:** Seyyed Mojtaba Mousavi, Gity Behbudi, Ahmad Gholami, Seyyed Alireza Hashemi, Zohre Mousavi Nejad, Sonia Bahrani, Wei-Hung Chiang, Lai Chin Wei, Navid Omidifar

**Affiliations:** 1 Department of Chemical Engineering, National Taiwan University of Science and Technology, Taipei City, Taiwan; 2 Department of Chemical Engineering, University of Mohaghegh Ardabili, Ardabil, Iran; 3 Biotechnology Research Center, Shiraz University of Medical Sciences, Shiraz, Iran; 4 Nanomaterials and Polymer Nanocomposites Laboratory, School of Engineering, University of British Columbia, Kelowna, BC V1V 1V7 Canada; 5 Nanotechnology & Catalysis Research Centre, University of Malaya, Kuala Lumpur, Malaysia; 6 Department of Pathology, Shiraz University of Medical Sciences, Shiraz, Iran

**Keywords:** Shape controlled zinc oxide nanostructures, Biomedical applications, Reactive oxidative stress, Macromolecules

## Abstract

Zinc nanostructures (ZnONSs) have attracted much attention due to their morphological, physicochemical, and electrical properties, which were entailed for various biomedical applications such as cancer and diabetes treatment, anti-inflammatory activity, drug delivery. ZnONS play an important role in inducing cellular apoptosis, triggering excess reactive oxygen species (ROS) production, and releasing zinc ions due to their inherent nature and specific shape. Therefore, several new synthetic organometallic method has been developed to prepare ZnO crystalline nanostructures with controlled size and shape. Zinc oxide nanostructures’ crystal size and shape can be controlled by simply changing the physical synthesis condition such as microwave irradiation time, reaction temperature, and TEA concentration at reflux. Physicochemical properties which are determined by the shape and size of ZnO nanostructures, directly affect their biological applications. These nanostructures can decompose the cell membrane and accumulate in the cytoplasm, which leads to apoptosis or cell death. In this study, we reviewed the various synthesis methods which affect the nano shapes of zinc particles, and physicochemical properties of zinc nanostructures that determined the shape of zinc nanomaterials. Also, we mentioned some macromolecules that controlled their physicochemical properties in a green and biological approaches. In addition, we present the recent progress of ZnONSs in the biomedical fields, which will help centralize biomedical fields and assist their future research development.

## Background

One of the most critical metal oxide nanoparticles is called Zinc oxide nanoparticles (ZnONPs), which is usually due to special physical and chemical properties are used in various fields. In personal care products, such as sunscreens and cosmetics, of the ZnONPs are increasingly used due to their strong UV absorption properties. Among the unique properties of ZnONPs are their UV blocking, antimicrobial and antibacterial properties [1, 2]. Controlling the shape and assembly of metallic nanostructures was used to regulate the catalytic, optoelectronic, magnetic, electronic, and optical biological efficiency of nanomaterials [3]. In addition to size, the shape of metal oxides and semiconductor crystals also profoundly affects their properties [4]. The size- and shape-dependent properties of nanocrystals can be adjusted by changing the synthesis medium and chemical intermediates involved in the synthesis process [5]. The synthesis of ZnO-controlled nanocrystals based on size and shape is done by adjusting growth parameters, including reaction time, source-to-pattern ratio Zn2þ, temperature and pH, and the type of material used in the pattern [6]. Recently, various techniques have successfully synthesized large quantities of ZnO nano and microstructures by different specific shapes [7]. Several new organometallic synthetic method has been developed to prepare ZnO crystalline nanoparticles with controlled size and shape [8]. Some of the shapes in which zinc oxide has been synthesized are nanorods, nanowires, nanobelts, and nanostars. One of the reasons for limiting the monodisperse solution syntheses with highly controlled shape for ZnO at the nanoscale is the same variation in morphology [9–12]. Shape-controlled zinc oxide nanostructures may affect its biocompatibility. For example, spherical zinc oxide nanoparticles (10–30 nm) have a more toxic effect on Ana-1 cells than the nanorod structure [13]. Among the factors that affect the antimicrobial capability of ZnO microstructures, the crystal size, composition, shape, crystal density, and morphology are more notable [14].

There are different ways to synthesize shape-controlled zinc oxide nanostructures (ZnONSs). The synthesis method majorly inclusive reflux of zinc acetate dehydrates (Zn (CH3COO)2.2H2O) as a procedure in organic solutions such as triethylene glycol (TEG) and diethylene glycol (DEG). Sodium acetate can have different morphology, size, and reaction time [15]. Various methods have been utilized to provision ZnONSs, such as the sol-gel technique and micro-emulsion synthesis [16]. Surface modification of NPs is done by linking macromolecules such as organic polymers and their derivatives. This method improves the polymer matrix, dispersibility of NPs in the polymer matrix, and causes to raising the characteristics of the resulting composites [17]. To achieve this goal, many macromolecules are used in the synthesis process of ZnONSs. This review will summarize synthesis methods to form shape-controlled ZnONSs, their physicochemical properties, macromolecules involved in forming nanoshapes of zinc particles, and recent progress of ZnONSs in the biomedical fields.

## Shape controlled synthesis methods

**Fig. 1:**
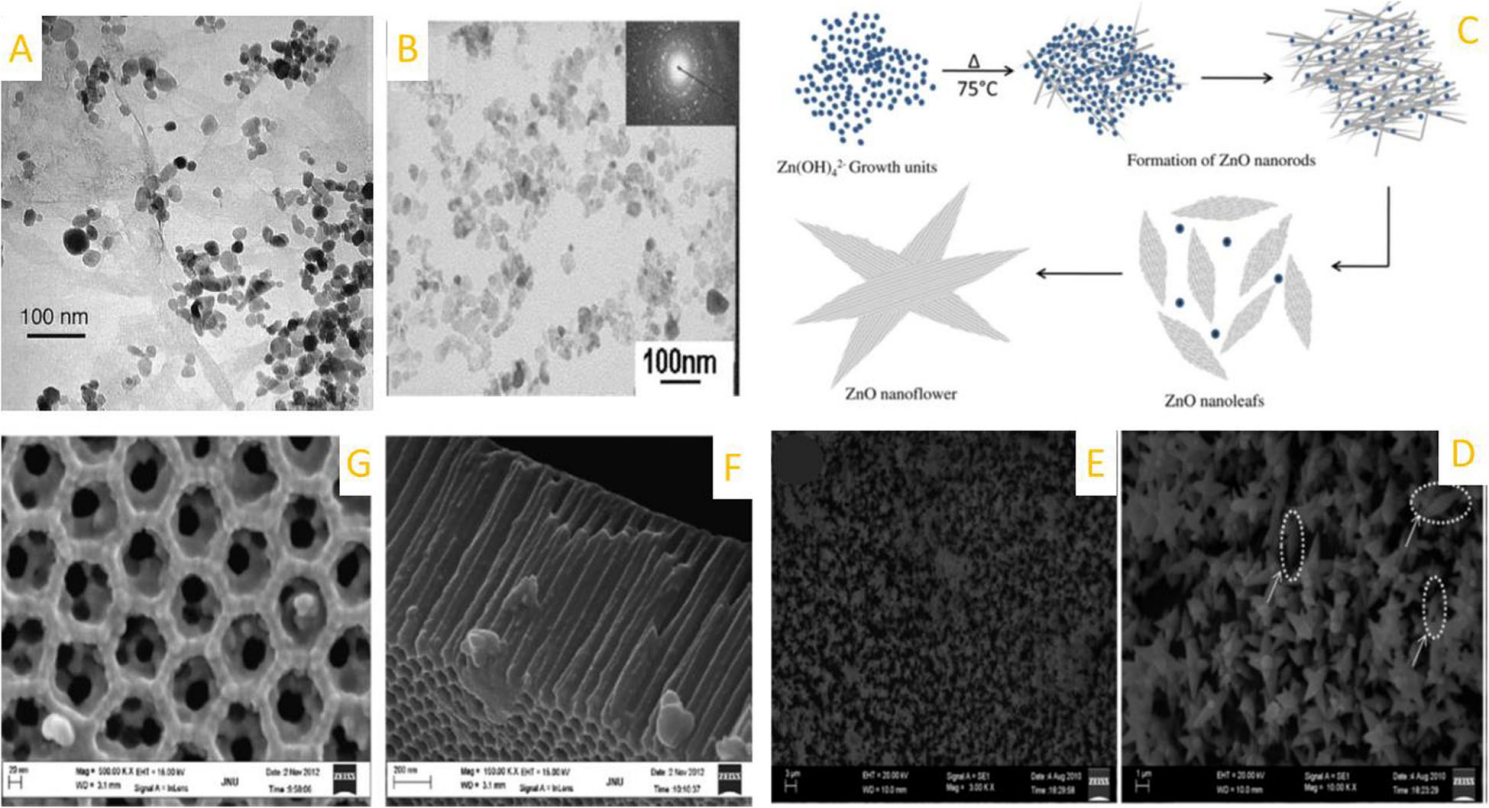
**A** TEM of ZnO nanostructures obtained by mechanochemical process [21], **B** TEM of ZnO using solvothermal method [22], **C**, **D**, **E** Formation mechanism of ZnO nanoflowers and its SEM micrograph synthesized by precipitation process [23], **F**, **G** SEM images of the ultrathin anodic aluminum oxide membrane after sol filling and annealing treatment in which ZnO nanotubes are injected by sol–gel into the pores of membrane [24]

**Fig. 2:**
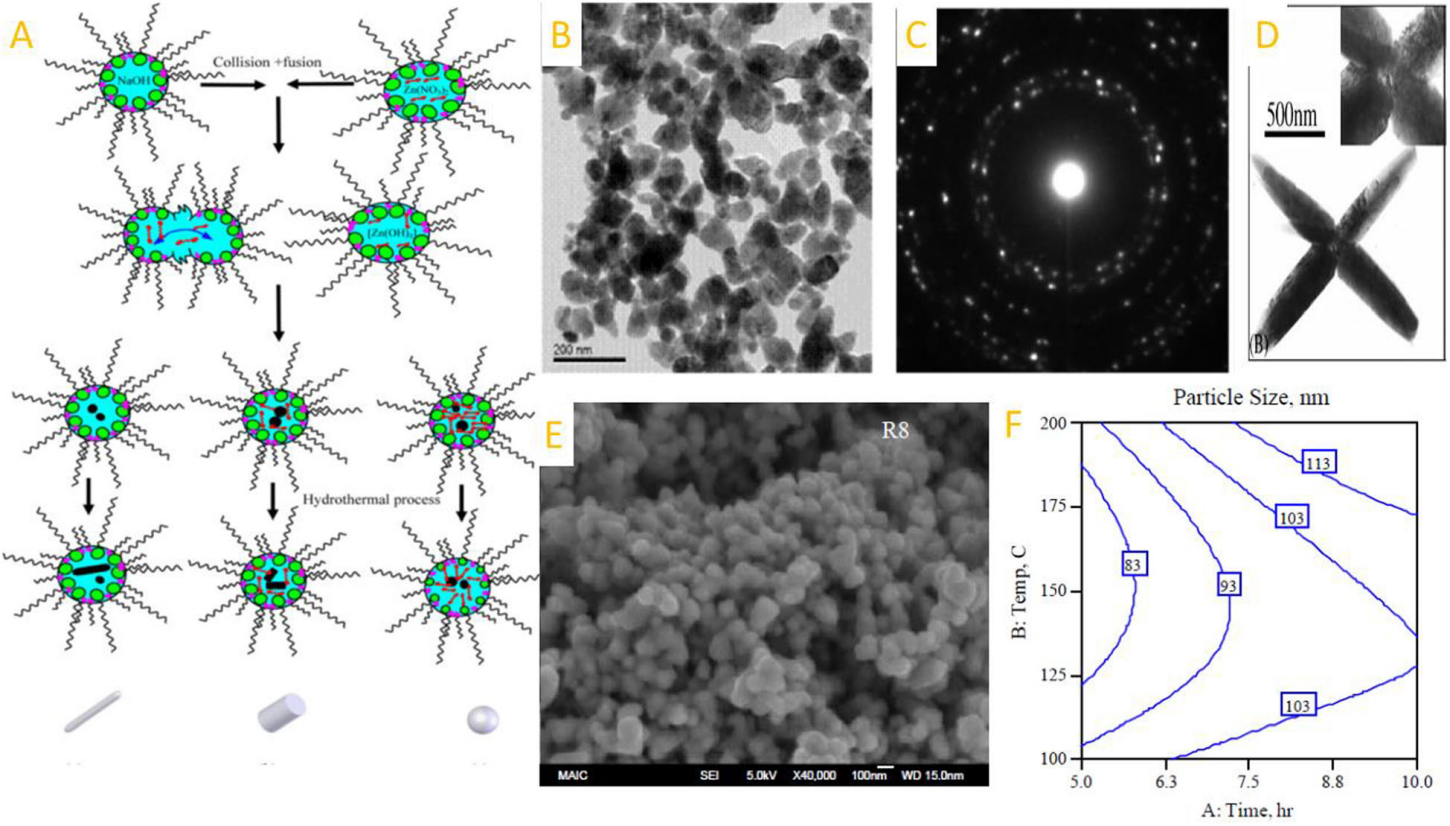
**A** Synthesis and morphology control of crystalline ZnO particles in microemulsions and **B**, **C** TEM and SAED patterns of ZnO nanoparticles calcined at 800 °C [25], **D** TEM of ZnO multipods using microwave-assisted mehode [26], **E**, **F** SEM and Contour plots for the effects of time and temperature on ZnO particle size using hydrothermal process [23]

**Fig. 3:**
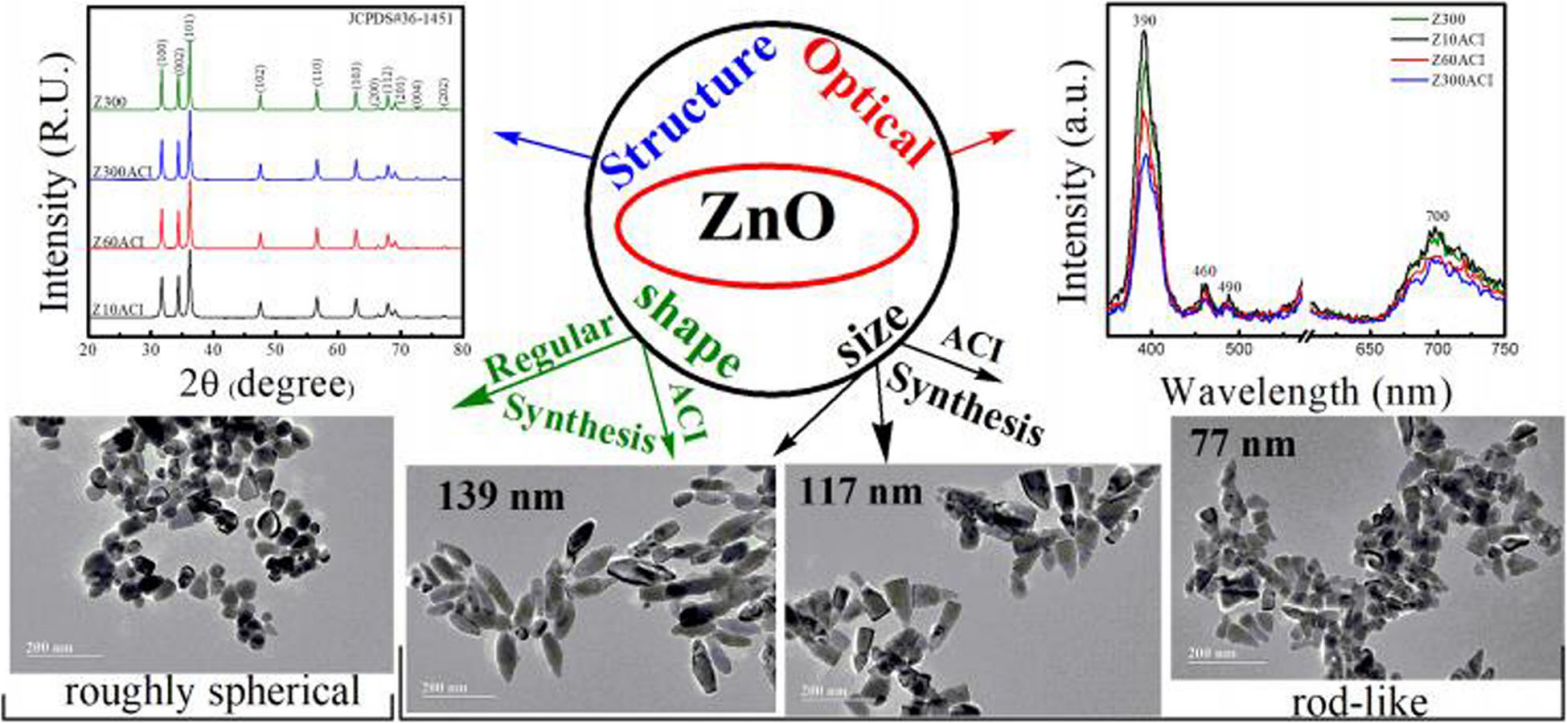
The effect of particle shape and size on the morphology and optical properties of shape-controlled synthesis of ZnO nanostructures [27]

A variety of shape-controlled ZnONSs, including nanowires, nanorings, nanotubes, and nano-tetrapods, are synthesized by different methods comprising chemical vapor deposition, electrodeposition, and thermal evaporation. These nanostructures have been subject to UV emission, electrical transport, gas sensing, and ferromagnetic doping investigations [18, 19]. In general, the methods for synthesizing zinc NPs are included solid-phase, liquid-phase, and gas-phase processes. The solid-phase techniques consist of mechanochemical processing, homogenous precipitation, the liquid-phase techniques include sol-gel technique, microemulsion synthesis, sonochemical synthesis, supercritical water processing, biosynthesis, and self-assembling, while the gas- phase processes comprise vapor transport process, spray pyrolysis, spray drying, microwave-assisted synthesis, thermal decomposition of organic precursors, radio frequency, plasma synthesis, hydrothermal processing, and hydrothermal processing Fig. 1. TEM shows the zinc nanoparticles obtained by chemical mechanical process and also Fig. 2 also shows the synthesis and morphology control of crystalline ZnO particles in microemulsions and TEM and SAED patterns and contour diagrams for the effects of time and temperature on the size of zinc oxide particles using a hydrothermal process [20]. The characterization of the different morphologies of shape-controlled of ZnO nanostructures can be performed via TEM, SEM, optical and XRD techniques. Figure 3 shows the effect of particle shape and size on the morphology and optical properties of shape-controlled synthesis of ZnO nanostructures. Table 1 is briefly depicted the synthesize method and the conditions that lead us to create the specific shapes of the nanostructure.

**Table 1:** Different synthesis conditions and methods for shape-controlled ZnONSs

Technique	Precursor	conditions	Features	Ref
Mechanochemcal process	ZnCl2, Na2CO3, NaCl	400–800 °C	hexagonal structure, 18-35 nm	[28]
		300–450 °C	51 nm particle	[21]
Precipitation Process	ZnSO4, NH4OH, NH4HCO3	2 h, 400 °C	hexagonal structure, flake-like, 60 nm	[29]
	Zn (CH3COO)2, NaOH	30 min, 75 °C	hexagonal structure, flower-like, 800 nm	[30]
	Zn (CH3COO)2, NH3 aq.	85 °C;	hexagonal structure, shape of rods, flower-like	[31]
	ZnCl2, NH4OH, CTAB	aging: 96 h, calcination: 2 h, 500 °C	zincite structure, 54-60 nm	[32]
Sol-gel	Zn (CH3COO)2, oxalic acid (C2H2O4), Ethanol	4 h at 650 °C	hexagonal wurtize structure	[33]
	Zn (CH3COO)2, diethanolamine, ethanol	2 h, 500 °C	hexagonal wurtize structure; nanotubes of 70 nm	[24]
Hydrothermal	Zn (CH3COO)2, NaOH, HMTA (hexamethylenetetraamine)	5–10 h, 100–200 °C	spherical shape, 55–110 nm	[23]
Microwave	Zn (NO3)2, deionized water, HMT (hexamethylenetetramine	2 min, 90 °C;	hexagonal wurtize structure, nanorod and nanowire shape (*L*: ~ 0.7 μm, *D*: ~ 280 nm);	[26]
Solvothermal	trimethylamine N-oxide, 4-picoline N-oxide, HCl, toluene, ethylenediamine (EDA), N,N,N′,N′-tetramethylethylenediamine (TMEDA)	24–100 h, 180 °C	wurtize structure; nanorods	[22]
Emulsion	Zn (CH3COO)2, heptanes, Span-80, NH4OH	aging: 2.5 h; calcination: 2 h, 700–1000 °C	hexagonal structure; spherical shape;, 0.05–0.15 μm	[34]
Microemulsion	Zn (NO3)2, NaOH, heptane, hexanol, Triton X-100, PEG400	15 h, 140 °C	hexagonal (wurtize) structure, needle (*L*: 150–200 nm, *D*: ~ 55 nm), nanocolumns (*L*: 80–100 nm, *D*: 50–80 nm), spherical (~ 45 nm)	[25]

### Vapor transport synthesis

The most usual technique for the synthesis of ZnO nanostructures is the vapor transfer process. In this procedure, zinc and vapor of oxygen react with each other, forming ZnO nanostructures. ZnO decomposition is a simple method to generate zinc and oxygen vapor. However, it requires very great temperatures (about 1400 °C). the other simple procedure is to heat the zinc powder under an oxygen stream. The benefit of this method lies in that the presence of graphite remarkably decreases the decomposition temperature of ZnO. Due to the differences in nanostructure formation mechanisms, the widely used vapor transfer process can be classified as catalyst-free vapor-solid process (VS) and catalyst-vapor-solid-vapor (VLS) process. Synthesis using the VS process is usually capable of creating a variety of nanostructures, including nanowires, nanotubes, nanorods. The use of ZnO nanostructures mainly relies on the capability to control their alignment, location, and packing density. Lithographic and non-lithographic patterning methods is used to control the locations of ZnO nanowires [35, 36].

### Sol-gel technique

Among the different methods, the sol-gel is one of the most promising methods to prepare ZnO nanoparticles. The sol-gel method is one of the easiest methods, controllable chemical composition, low temperature of decomposition, low cost, energy efficiency, high rate of production, and synthesis of NPs. These advantages make the sol-gel technique a very attractive preparation method. In this method, the size and morphology of the particles can be controlled by adjusting the reaction parameters [37]. Hasnidawani et al. synthesized ZnONSs by the sol-gel method. In this method, Zinc acetate dehydrates (Zn (CH_3_COO)_2_.2H_2_O), ethanol (CH_2_COOH), Sodium hydroxide (NaOH), distilled water were used as a precursor, solvent, and medium, respectively. ZnONSs had range sizes of 81.28 nm to 84.98 nm. FE-SEM results showed that ZnONSs had a structure like a rod ZnO and, according to XRD results, crystalline properties [16]. Abdullah et al. synthesized ZnONSs by the sol-gel method. They mixed methanol solution and Zinc acetate dehydrate. Ammonia NH_4_OH was used to adjust the pH of the solution in the range of 9 and 11. ZnONSs had the range sizes of 12 nm to 30 nm. Atomic force microscopy (AFM) results showed that ZnONSs had homogenous [38]. Jurablu et al. synthesized ZnONSs by the sol-gel method. They used ethanol solution, zinc sulfate heptahydrate, and diethylene glycol as the surfactant. The average size of ZnONSs was 28 nm. Also, according to the results, ZnONS have a hexagonal wurtzite structure [39]. Alwan et al. synthesized ZnONSs via the sol-gel method. In this method, zinc acetate dehydrates and distilled water is used as precursors and solvent. Alcohol and hydrogen peroxide were then added to the solution and heated. XRD results showed that ZnONSs had a wurtzite crystal structure, and the Fourier-transform infrared spectroscopy (FTIR) absorption band appeared at 417.52 cm^− 1^_._ SEM results indicated that ZnONSs had a spherical shape and smooth surface with an average size of 100–200 nm [40]. Kumar et al. synthesized ZnONSs by the sol-gel method. Sodium Hydroxide and Zinc Acetate (ZnAc) were added to polyethylene Glycol (PEG). PEG and ZnAc were utilized for the establishment of Zn-alkoxide. By heating the mixture at 80 °*C* until it reaches boiling temperature, at last, this sol could turn from a sol mood into gel mood, and high thickness gel was created. For creating ZnONSs, HNO_3_ was added to make pH acidic, then the solution was filtered and dried, and in the following by calcination, ZnONSs formed [41]. The morphologies of ZnONSs obtained in different solvent systems via a sol–gel synthesis shown in Table 2.

**Table 2:** The morphologies of ZnONSs via a sol–gel synthesis [42]

Morphology	Solvent	Ref.
Very short hexagonal rods	m-Xylene: H_2_O	[42]
Globular shaped particle-like structures	Hydroquinone: H_2_O	[42]
A mix of wide slates and thin hexagonal rods	Toluene: H_2_O	[42]
Thin slates like structures	DMSO: H_2_O	[42]
Hexagonal rods	Acetonitrile: H_2_O	[42]
Hexagonal rods	DMF: H_2_	[42]
Hexagonal wurtzite structure	Ethanol: H_2_O	[39]
Rod-like structure	Ethanol: Sodium hydroxide (NaOH) and distilled water	[16]
Thorn like morphology with wurtzite crystal structure	Cetyltrimethylammonium bromide: H_2_O	[43]
Highly crystalline, having wurtzite crystal structure, spherical in shape with smooth surface	Alcohol: Distilled water	[40]
Wurtzite structure	Citric acid: H_2_O	[44]

### Microemulsion synthesis

For preparing particles with low particle size polydispersity and average diameters smaller than 10 nm, microemulsions is a well-established technique [45]. This synthesis method does not require any rigorous experimental conditions, sophisticated equipment, and complex preparation procedure, but still providing possibilities in controlling the morphology of the ZnO powders and size in a size scale approaching to nanometers. The superior aspect of the ZnONSs obtained by microemulsion routes is achieving narrow size distribution due to well-dispersed cage-like small reactors (5–100 nm) formed in uniform nucleation conditions [46]. Bumajdad et al. synthesized ZnONSs by the water-in-oil micro-emulsion method. The heptane, water, and surfactants system consist of 90% molar di-dodecyl dimethylammonium bromide (DDAB) and 10% molar Brij 35 created a microemulsion system. In this technique, head groups and Br − counter-ions of cationic surfactant interacted with OH^−^ and Zn^2+^ ions and could control the ZnONSs characteristics and nucleation process. ZnONSs had high surface area and porosity [47]. He et al. Synthesized ZnONSs in microemulsion and controlled the morphology. In this method, PEG400 was used as a directing agent. The oil phase consisted of hexanol and heptane (mol ratio of 1:3), and the polyoxyethylene tert-octylphenyl ether (Triton X-100) was used as the surfactant. Results showed that ZnONSs in the micro-emulsion system showed good controllable bandgap energy, morphology, and crystalline size [25]. Sanchez et al. synthesized Zn NPs doped with TiO2 in the microemulsion method and were used in phenol photodegradation. Zn-doped TiO2 NPs showed photocatalytic activity due to the production of oxygen lacuna in the surface and creating tetracoordinate Ti on nanosized particles. Oxygen lacuna in the surface caused to decreasing in charge of the electron and increasing OH and chemisorption of oxygen. Creating tetracoordinate Ti on nanosized particles caused increasing high dispersity, reducing particle size, and rising photocatalytic activity [48]. Yıldırım et al. synthesized ZnONSs by micro-emulsion method from sodium bis (2-Ethylhexyl) sulfosuccinate (Aerosol OT)/ glycerol/ heptane. Zinc acetate dehydrates, Aerosol OT, glycerol, and n-heptane were used as a procedure, surfactant, polar phase, and non-polar phase, respectively. The presence of Aerosol OT, glycerol and heptane caused to formation microemulsion system. In this system, the ZnONSs formation was occurred due to the calcination of zinc glycolate under high temperatures. The size and morphology of ZnONSs were affected by Aerosol OT concentration in the microemulsion and the calcination temperature. The low and high concentrations of surfactant caused spherical and rod-like ZnONSs, respectively. With increasing the temperature of calcination, the average size of NPs was increased [46]. Shape and size of ZnONSs obtained from TEM images in in water-in-oil microemulsion synthesis shown in Table 3.

**Table 3:** Shape and size of zinc oxide nanoparticles [49]

Shape	Conc.	Ref.
Rectangular structures	Sodium hydroxide (0.525), Zinc (0.25)	[49]
Rods/wires	Sodium hydroxide (0.225), Zinc (0.10)	[49]
Spherical	Ammonium hydroxide (1.0), Zinc (0.1)	[49]
Spherical	Ammonium hydroxide (1.0), Zinc (0.4)	[49]
Spherical	Ammonium hydroxide (1.0), Zinc (0.25)	[49]
Spherical	Ammonium hydroxide (1.0)), Zinc (0.1)	[49]
Hexagonal wurtzite structure	12.5 and 25.0% (wt%) PEG400 in Zn (NO3)2 solution	[48]
Spherical	5:5:90 (AOT:glycerol:n-heptane) in weight percentages	[46]
Rod like	30:5:65 (AOT:glycerol:n-heptane)	[46]

## Chemistry and physicochemical properties of ZnNSs

**Fig. 4:**
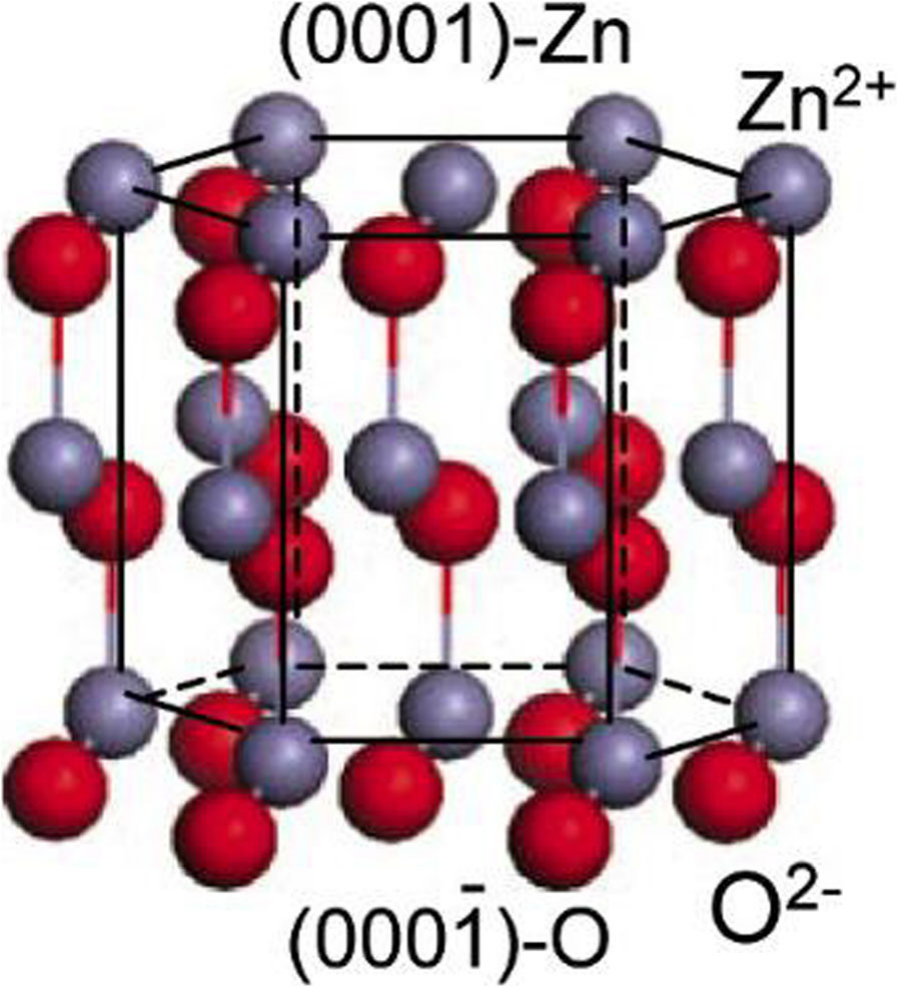
The structure of ZnO [51]

One zinc atom is tetrahedrally coordinated with four oxygen atoms in the Zinc oxide structure. Such tetrahedral coordination of ZnO gives rise to the noncentrosymmetric structure, which is attributed to the piezoelectric nature [50]. Figure 4 shows the structure of ZnO that consists of O_2_^−^ and Zn^2+^ ions and is located tetrahedrally.

**Fig. 5:**
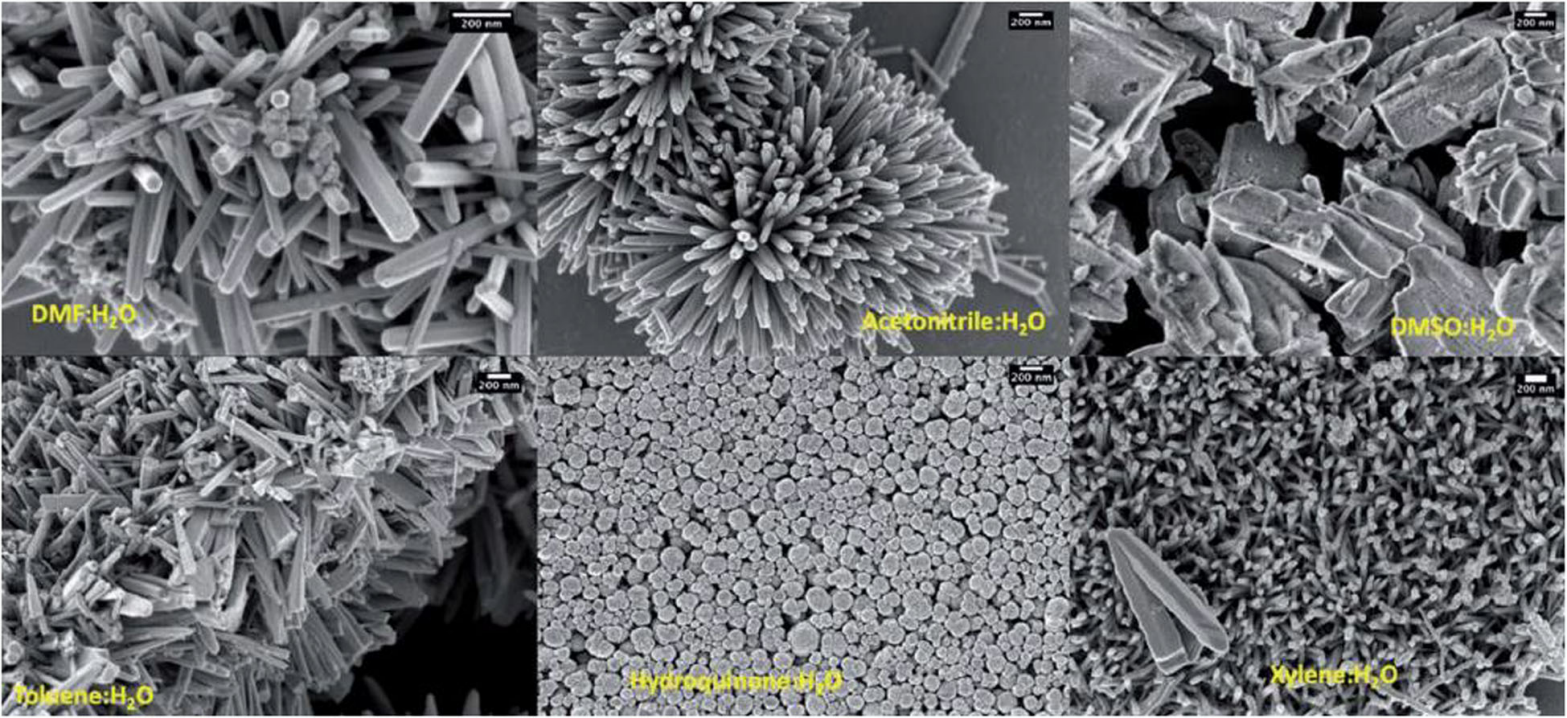
SEM of ZnONSs [53]

ZnONSs have been mainly considered because of achieving some characteristics in nanoscale, such as the large ratio of area to volume, wide energy band gap, ease of building, affordable economic synthesis and, environmentally friendly nature [52]. Scanning electron microscopy (SEM) of synthesis zinc (II) chloride with NaOH in different solvents has been shown in Fig. 5. The images indicate different morphologies containing hexagonal nanorods, slate-like structures, and globular-shaped nanostructures; Hexagonal nanorods were formed in dimethylformamide aqueous solution, and irregular slate-like ones were constructed in dimethyl sulfoxide solution. Globular-shaped nanostructures were formed in hydroquinone and water. The type of solvent could also affect the length of nanostructures [42].

**Fig. 6:**
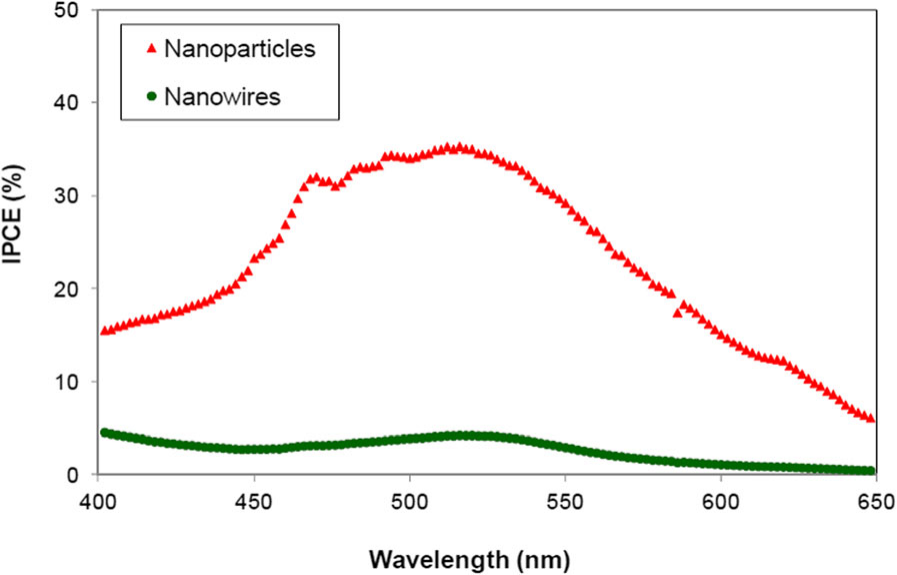
IPCE values of nanoparticles and nanowire of DSSCs as a function of wavelength [18]

Giannouli et al. compared the function of nanowire films and nanoparticle-based ZnO dye-sensitized solar cells (DSSCs) to investigate the factors that affected power conversion efficiency in ZnO DSSCs. NPs and nanowire arrays showed 6.2 and 0.63% efficiency, respectively. According to Fig. 6, dye loading in nanowire films was fewer than that of NPs, due to repelling electrostatic forces between dye and the nanowire surface [18].

**Fig. 7:**
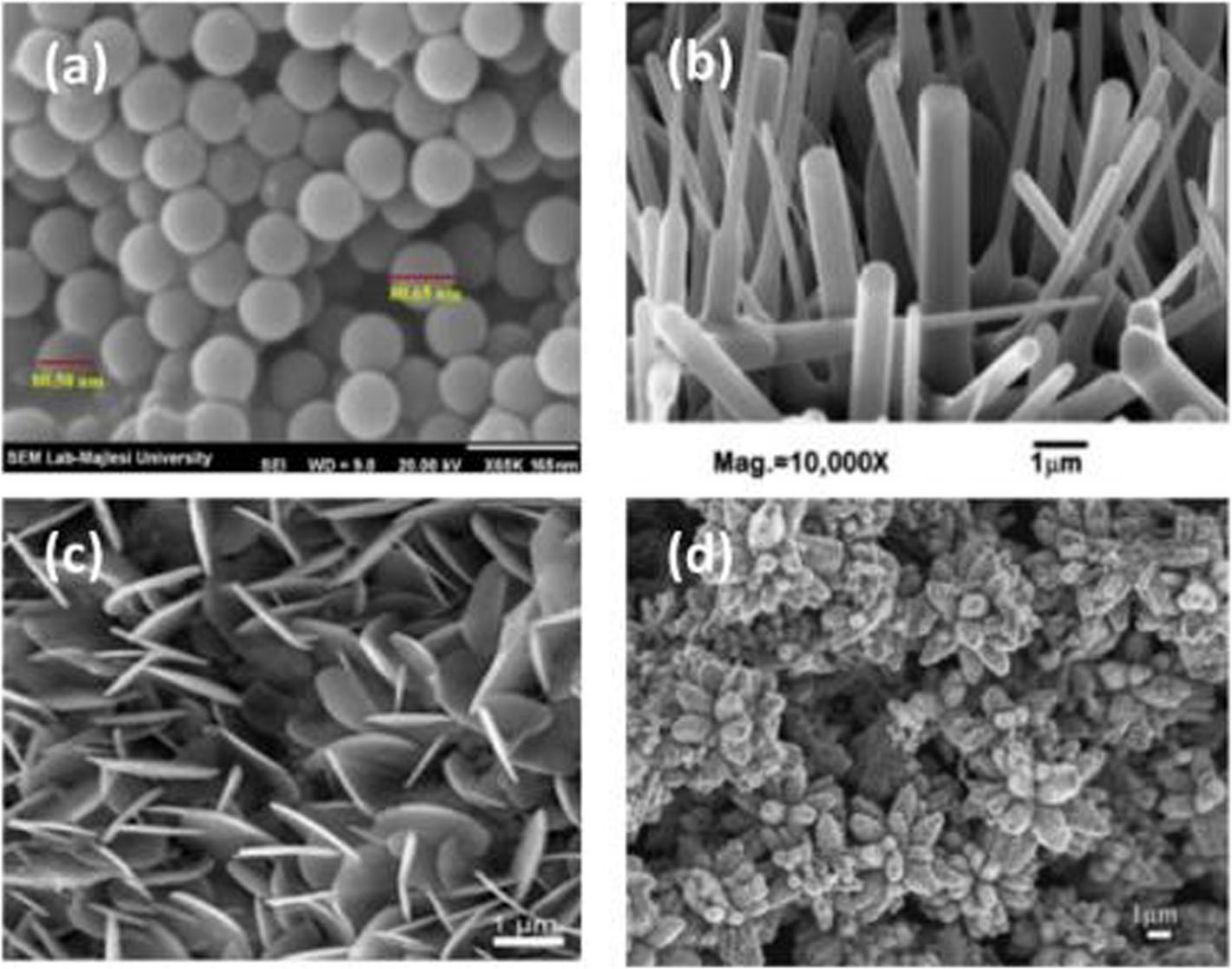
Morphologies for **a** 0D, **b** 1D, **c** 2D, **d** 3D shape-controlled of ZnO nanostructure [58]

These physicochemical properties of shape-controlled ZnONSs are influenced by the synthesis techniques used for their preparation [54]. The morphology of the shape-controlled ZnONSs with different dimensions is shown in Fig. 7. Nanorods, nanofibers, nanowires [55, 56], nanotubes [57], and nanoneedles are demonstrated in 1D shape-controlled ZnONSs arrays. Examples of shape-controlled ZnONSs can be seen in 2D and 3D arrays of nanosheets and nanoflowers, respectively.

ZnO nanoparticles have extravagant physical properties. Some of the physical properties of semiconductor materials undergo changes known as “quantum size effects” as the dimensions continuously decrease to the nanometer. For example, bandgap energy of quasi-one-dimensional (Q1D) ZnO increased by quantum confinement, confirmed by photoluminescence [59].

## Macromolecules used in the synthesis of zinc NSs

Surface modification of NPs creates a link between polymers and their surface [60, 61]. This method improves the polymer matrix, dispersibility of NPs in the polymer matrix and causes to raising the characteristics of the resulting composites [17]. Different macromolecules can synthesize ZnONSs such as PEG and cellulose derivatives [62] described below.

### Peg

PEG can be adsorbed to the surface of metal oxides and alter the kinetics of growing colloids. The viscosity also increases with the dissolution of polyethylene glycol in ethanol [63]. This procedure reduces the preparation time of the ZnO and increases the amount of crystallization of ZnONSs. Since the PEG is added to the ZnO, the amount of electrostatic adsorption of ZnONSs has increased [17]. Hou et al. investigated the synthesis of ZnONSs in the presence of short-chain polymer PEG solutions with different concentrations to synthesize ZnO nanostructure. An excess amount of NaOH was dissolved in the ZnAc solution. The achieved solution was added to different concentrations of the PEG solution. After centrifugation and washing with ethanol and water, white crystalline ZnONSs were collected and dried in a vacuum. Results showed that a small PEG could produce ZnONSs with rod-like and needle-like, whereas a small PEG could produce ZnONSs with flower-like structures [64]. Tshabalala et al. synthesized ZnONSs with PEG using the sol-gel method. ZnAc was used as a procedure, and by using PEG in a chemical method and distilled water or ethanol as solvent, ZnONSs were formed.

The average diameter of NPs was 40–50 nm. The photoluminescence emission observed 325 nm, which was used for excitation. The useful modality of ZnONSs showed the green and blue luminescence. Nanocrystals had a high density of surface states because of the high surface-to-volume ratio of NPs and the small crystals [17]. Leila et al. investigated the effect of propyltrimethoxysilane (PTMS) and PEG as a surfactant on the properties of ZnONSs that were synthesized by the sol-gel method. PTMS and PEG together could guard the surface against agglomeration, photoemission properties, crystallinity and, control of the size. In this method, ZnONSs had a hexagonal wurtzite structure, a small size of about 6 nm, and isotropic crystal properties. The photoluminescence observed at 390 nm showed blue emission related to the oxygen vacancy [17]. Shape and size of ZnO NPs obtained using PEG synthesis as macromolecule shown in Table 4.

**Table 4:** Shape and size of zinc oxide nanoparticles with PEG

Shape	Amount	Ref.
Wurtzite or hexagonal structure	1 g zinc nitrate hexahydrate Zn (NO3)2 ·6H2O and 0.3 g PEG (6000) and PVP with	[65]
Rod-like and plate-like crystals	0.25 M Zn (NO3)2.6H2O, 5 M NaOH (1:20 M ratio of Zn:OH) and PEGs of high molecular weight (1500 and 4000) were added in excess amount at 10 and 25 wt%	[66]
Nanowires	Zn (CH3COO)2â2H2O (1.100 g, 5.02 mmol) and 5 mL of PEG400	[22]
Nanorods	Zn (CH3COO)2â2H2O (0.4522 g, 2.06 mmol) and 5 mL of PEG400	[22]

### Cellulose derivatives

Cellulose is in natural polymers and is one of the most abundant polymers that contain a large specific area [67]. Because of high surface energy and large surface area, ZnONSs have aggregation ability. While synthesizing ZnONSs with nanofibrillar structures such as cellulose can overcome this problem [68]. The combination of cellulose as a semiconductor in metal NPs composites causes ion absorption valence, biocompatibility, sensitivity, high thermal stability, and nontoxicity properties [69]. Mocanu et al. synthesized ZnONSs in the presence of bacterial cellulose (BC) through the ultrasound method. The BCZnO is medicated with an ethanolic extract of propolis. For synthesizing ZnONSs, ammonia was added to the solution of zinc acetate, BC, and distilled water, to raise the pH until 11. Then the solution was sonicated in an ultrasound bath [70, 71]. Ali et al. synthesized zinc nanocomposites medicated with cellulose separated from the waste of citrus peel. ZnO medicated with cellulose showed significant destruction of batteries exclusivity, increasing the time of degrading methylene blue and antioxidant activity compared to ZnONSs. The size of ZnONSs was 50 nm. For producing ZnONSs, cellulose fibers were swallowed in 1 mM zinc acetate dihydrate (Zn (CH3COO)2.2H2O) under stirring, and then the solution was sonicated. Treating the solution with 0.01 M Sodium hydroxide (NaOH) caused a reduction of zinc into nanoparticles [69]. Bagheri et al. synthesized ZnO with cellulose nanocomposite by the microwave-assisted hydrophilic ionic liquid. 1-butyl-3-methylimidazolium chloride ([C4mim]Cl) was used to dissolve cellulose and synthesize ZnONSs. This work dissolved cellulose in ([C4mim]Cl) under microwave plus. Then ionic liquid with sodium hydroxide was mixed with zinc acetate. The mixture was stirred with distilled water and the composite separated from ionic liquid.

At last, the product was dried [67]. Yu et al. synthesized ZnO nanohybrids with cellulose nanocrystal (CNC) by acid hydrolysis of cellulose and precipitation with zinc nitrate solution for photocatalytic and antibacterial application. ZnONSs had 42.6 nm. CNC-ZnO indicated photocatalytic and antibacterial activity for methylene blue because of strong interactions among cellulose nanocrystal and ZnONSs [68]. Shape and size of ZnO NPs obtained using cellulose derivatives as macromolecule shown in Table 5.

**Table 5:** Shape and size of ZnO NPs obtained by using cellulose derivatives as macromolecule

Shape	Type of ZnONPs	Ref.
Spherical	Carboxymethyl cellulose (CMC) capped Ag-ZnOnanoparticles (NPs)	[72]
Spherical	Hydroxyethyl Cellulose	[73]
Rod-shaped	ZnO-overlaid cellulose nanocrystals (CNCs)	[74]
Hexagonal wurtzite structure	Cellulose–ZnO-hybrid nanocomposite	[75]

### Biomedical applications of different shape-controlled ZnONSs

**Fig. 8:**
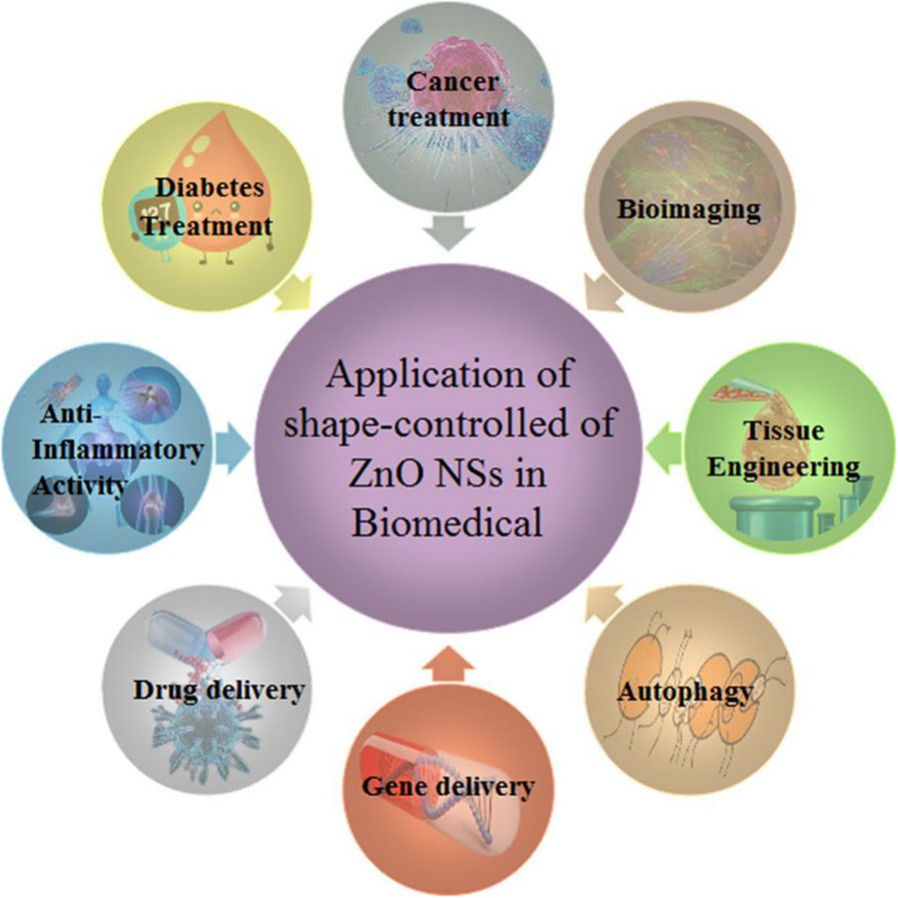
Biomedical applications of shape-controlled ZnONSs

As shown in Fig. 8, shape-controlled ZnONSs have emerged a promising potential in biomedicine, especially in the fields of cancer treatment, diabetes treatment, inflammatory activity, drug delivery, gene development, and autophagy activity, which are involved with their potent ability to trigger excess ROS production, release zinc ions, and induce cell apoptosis [76–78]. ZnONSs has electrical properties and has applications in semiconductors. Because of owning pharmaceutical properties, ZnO could use sunscreens and lotions [2, 79]. They have the high ability to absorb ultraviolet rays and repel dangerous waves from the skin. Therefore, they are a good option for making sunscreens [80]. Also, ZnONSs own extraordinary electronic, optical, mechanical, magnetic, chemical, and antibacterial properties [59].

ZnONSs less than 100 nm are considered to be relatively biocompatible, which represent a potent property in promoting the biomedical and research support their biomedical applications. Here, we summarized the recent progress on zinc NSs in biomedical applications [81].

### Cancer treatment

NSs are used to diffuse in several cancer cell lines including, malign human gliomas and hepatocarcinoma cell lines [82, 83], and inhibit the cancer cell overgrowth via caspase-dependent pathways and oxidative stress. Also, NPs less than 100 nm are very efficient in animal studies [63, 84, 85]. Some shape-controlled ZnONSs can impact many cancers in vitro because of producing reactive oxygen species (ROS) by Zn ^2+^. Using ultraviolet light (UV), ZnO electrons can be transferred from the valence band to the conductive band, resulting in photocatalytic ROS production [76, 86]. Cancer cells can become toxic when Zn^2 +^ ions are released into the ZnO solution. ZnONSs can detriment the DNA via oxidative stress in epidermal cells of the human body and chain reaction of lipid peroxidation [87].

Ou et al. used core-shell structured zinc porphyrin (ZnONS) with polydopamine (PDA) to improve photothermal or photodynamic cancer therapy. This method manufactured the core-shell structure via near-infrared (NIR) by encapsulating the (ZnONSs) and PDA. ZnONS@PDA NPs were biodegradable and did not aggregate after injection. ZnONS@PDA NPs had a 46.8% photothermal conversion efficiency (PCE). Results showed that ZnONS@PDA NPs could tumor-targeting through enhanced permeability and retention (EPR) effect, leading to prominent phototherapy of tumor effects and powerful phototoxic properties. PDA photothermal proficiency had a high photothermal conversion efficiency of about 46.8% since the fluorescence of ZnONS could impressively be quenched by the shell of PDA through fluorescence resonance energy transfer (FRET) because of the existence of an overlap between sorption of PDA and transpiration of the ZnONS. Also, ZnONS@PDA had significant photodynamic effects and were the hopeful phototherapeutic agents for cancer therapy [88]. Wahab et al. surveyed the impact of ZnONSs for treating cancer cells. Results showed that ZnONSs were valid on cancer cells such as lung cancer cell line NCI-H460, human glioblastoma T98G cells, and thyroid cancer cells in SNU-80, and less poisonous to benign medical research council cell strain 5 (MRC-5) and human embryonic kidney 293 (HEK293) cells. Shape-controlled ZnONSs could form oxidative stress by generating ROS, damaging human cancer cells, and having considerable inhibitory efficacy in developing glioma cells. Over time, glioma cells became less viable, which showed that oxidative stress of ZnONSs could lead to apoptosis or cell death [87]. Tanino et al. investigated the anticancer role of ZnONSs versus small-cell lung cancer in vitro. Results showed that it was possible to inject ZnO intravenously against orthotopic small-cell lung cancers, with no side effects. Also, ZnONSs had no toxic effect versus small-cell lung cancer and incited the ROSs [76].

### Diabetes treatment

Metals are involved in glucose metabolism, and their deficiency leads to diabetes [89]. Zinc helps to maintain blood sugar and is effective in treating diabetes. Zinc is one of the essential elements and micronutrients needed by the body, which plays a vital role in activating the body’s enzymes, glucose metabolism, and cellular processes, including oxidative homeostasis, apoptosis, and immune function. Zinc improves hepatic glycogenesis during its activities in the insulin pathway, thereby modifying glucose utilization. Zinc preserves the structure of insulin and plays a role in storage, biosynthesis and, insulin secretion [90–92].

The presence of zinc leads to enhanced glucose transport into adipose tissue and skeletal muscle, reducing circulating glucose levels, intestinal glucose uptake inhibition, decreasing glycogenolysis and gluconeogenesis, improving the structural integrity of insulin, and improving insulin signaling [93]. Another study shows the antidiabetic role of green synthesized ZnONSs. ZnONSs have been synthesized by using plant extract. The reaction of NPs formation was under heating. The results of this study showed that small-sized of NPs indicated superior antidiabetic efficacy on diabetic mice rather than large-sized of NPs. Real-time polymerase chain reaction showed that ZnO could compel and expressions of pancreas and insulin receptors and the operation of T helper cells [94].

Alkaladi et al. investigated the antidiabetic role of ZnO and silver NPs on streptozotocin-induced diabetic rats. ZnO and silver NPs caused fasting serum insulin, lower blood glucose, higher insulin expression level, higher activity of glucokinase, increased insulin receptor, higher in the glucokinase gene, and glucose transporter 2. In this work, shape-controlled ZnONSs and silver NPs acted as antidiabetic drugs. However, ZnONSs were much more potent than silver NPs [90]. Siddiqui et al. studied the effect of ZnONSs versus diabetes diseases in mice. They surveyed oral glucose tolerance test (OGTT), hypoglycemic and antidiabetic roles of ZnONSs. Results showed that ZnONSs reduced blood glucose levels to 39.79%, whereas the blood glucose reduction for injection by insulin and the combination of insulin and ZnONSs were 48.60 and 38.78%, respectively. Hypoglycemic studies showed that 8 and 14 mg/kg of body weight amount of ZnONSs could reduce blood glucose levels to 25.13 and 29.15%. OGTT tests also confirmed the observed reductions in blood sugar [92]. Umrani et al. evaluated the antidiabetic role of ZnONSs in diabetes type 1 and 2 induced by streptozotocin. Results demonstrated that using ZnONSs led to antidiabetic effects, which caused 48% lower triglyceride levels, 70% higher levels of serum insulin, 40% reduction of non-esterified fatty acids, and 29% low blood glucose levels, and improvements in glucose tolerance [93]. Othman et al. studied the role of ZnONSs in microRNAs disorders in STZ-induced type 2 diabetes. Results showed that 5 mg/kg of body weight of ZnONSs caused increasing glucose tolerance, improving blood insulin status and function of pancreatic beta cells [95].

### Antibacterial activity

**Fig. 9:**
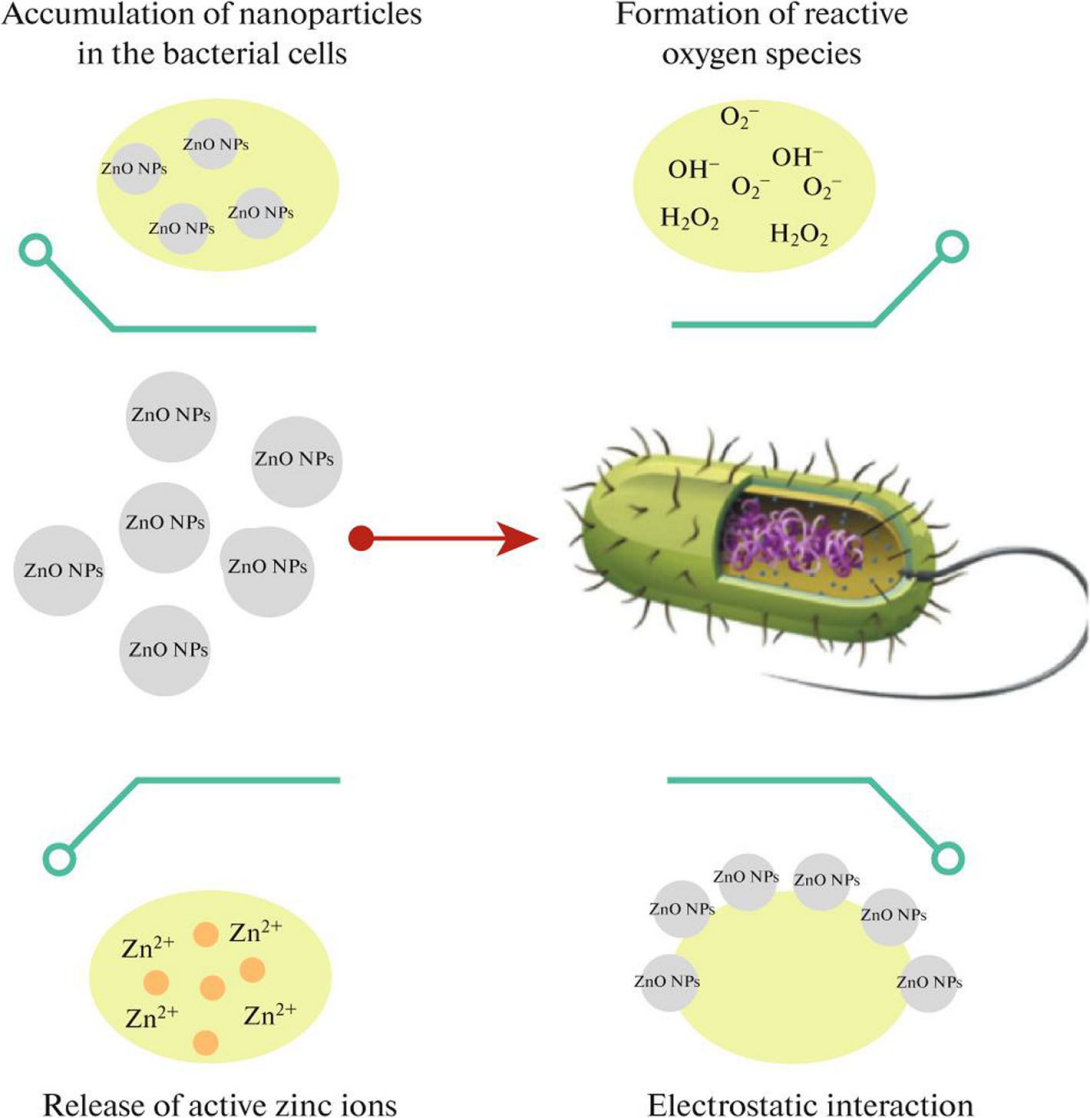
Mechanism of antibacterial activity of ZnONSs [18]

The shape-controlled ZnO materials’ physicochemical properties mainly affect their antimicrobial activity against pathogenic microorganisms and are vital parameters related to pharmacological and toxicological responses [96, 97]. The development of superior antimicrobial activity in pathogenic microorganisms is determined by morphology, particle size, and porosity of shape-controlled ZnONSs [98]. According to Fig. 9, ZnONSs can interact electrostatically with the bacterial cell wall and destroy the bacterial cell [53]. Cationic charged zinc ions with accumulate in the bacteria membrane cell and form reactive oxygen species [53, 99, 100].

Several shape-controlled ZnONSs at concentration of 100 mg/L can produce reactive oxygen species and show antibacterial activity in pH equal to 7 [79]. Prociak analyzed physicochemical properties of antimicrobial compositions treatment of surfaces based on polyvinyl alcohol and ZnONSs (3% concentration). The size of zinc oxide in the final composition was 232–692 nm. ZnO showed good antimicrobial properties and powerful adhesion forces [2].

Amna synthetized a ZnO nanoflowers thorough hydrothermal method and indicated considerable antibacterial activities against *E. coli* and controlling the growth of representative food pathogens within a short time and at low concentrations. In the future, cost-effective methods such as nanoflowers will be used as food preservatives and antibiotics because they are efficient and non-toxic [101].

### Anti-inflammatory activity

**Fig. 10:**
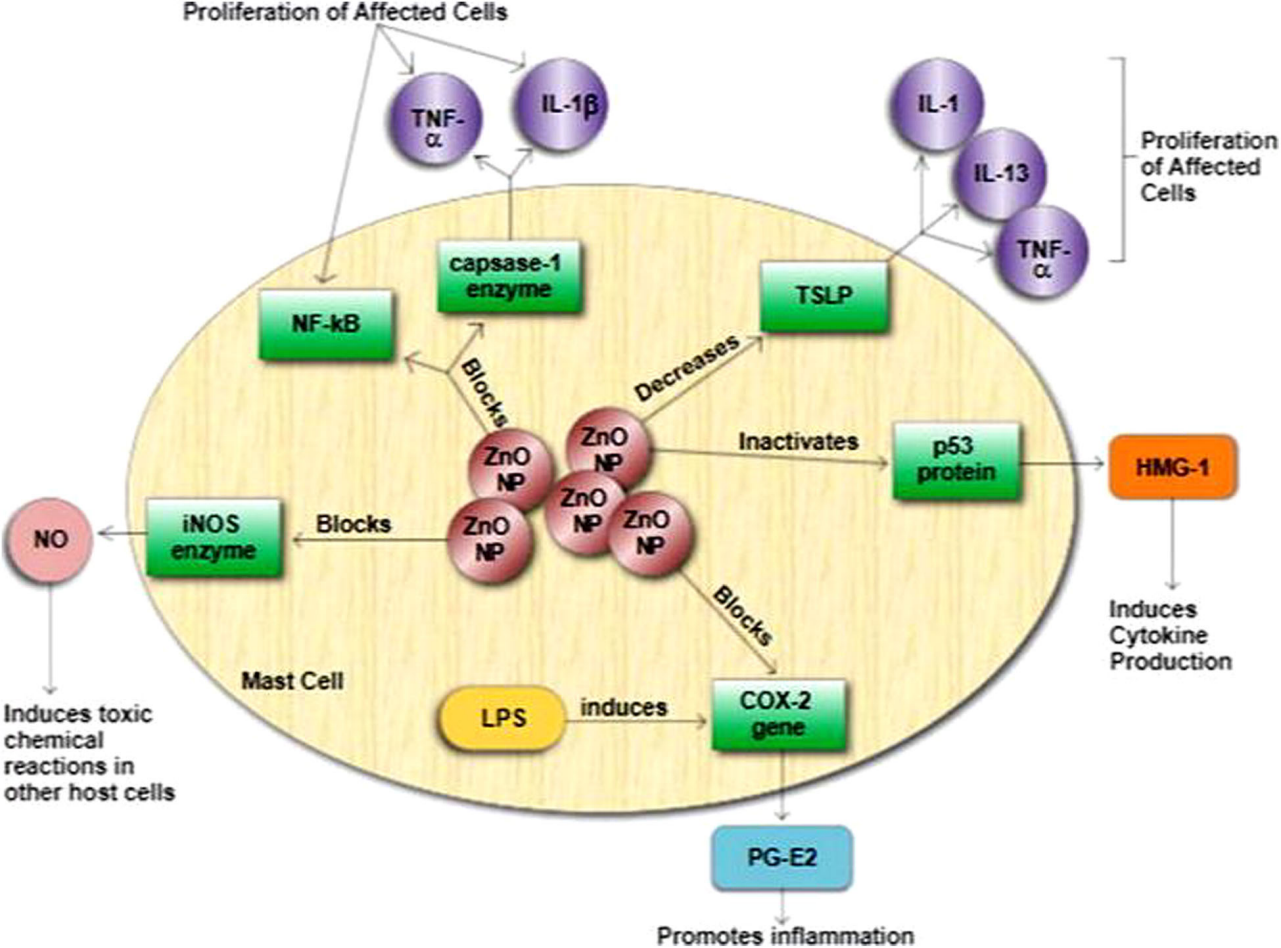
Mechanism of anti-Inflammatory activity of ZnONSs [103]

The response of inflammatory activity is an intricate procedure, which consists of the release of chemicals by the immune system such as Interleukin (IL)-18, interferon, tumor necrosis factor, IL-1, IL-12, and IL-6 cytokines and the operating white blood cells [102]. Mechanisms used by ZnONSs to exhibit anti-inflammatory activity include inhibition of release of the proinflammatory cytokine, inhibition of expression of inducible nitric oxide synthase (iNOS) enzyme, inhibit the NF-κB signaling pathway, inhibition of mast cell degranulation, and inhibition of myeloperoxidase (Fig. 10) [103]. ZnONSs can decrease thymic stromal lymphopoietin (TSLP) production. TSLP is sprinkled under physical hurt or stress situations. The most common mechanism of ZnONSs for showing anti-inflammatory activity is repressing inflammatory genes like IL-1, IL-13 and, TNF-α. ZnONSs inactive p53 protein and high mobility group box 1 (HMG-1) protein. HMG-1 can activate p53 protein DNA. Lipopolysaccharide induces PGE2 and cyclooxygenase-2 (COX-2) expression production. ZnONSs can block the COX-2 gene and prostaglandin E2, the most important cause of symptoms of inflammation. ZnONSs can block iNOS enzymes. iNOS can produce nitric oxide, which causes inflammatory disorders. The expression of TNF-α and IL-1β is the most usual mechanism by ZnONSs for showing anti-inflammatory activity [103].

Nagajyothi et al. investigated the anti-inflammatory role of ZnONSs produced with green synthesis by using the roots of Polygala tenuifolia. Anti-inflammatory activity was surveyed in Macrophage RAW 264.7 cells, stimulated with Lipopolysaccharide. ZnONSs showed superior anti-inflammatory activity by protein expressions and mRNA in a dose-dependent manner of TNF-α, iNOS, IL-6, IL-1b, and COX-2 [102]. Mobarez et al. investigated the anti-inflammatory efficacy of green synthesized ZnONSs from the olive plants on dexamethasone. Results showed that ZnONSs affected the Th2, which was enhanced by dexamethasone. ZnONSs had an indirect suppressive impact on the humoral immune-mediated by Th2 instead of directly affecting B-lymphocytes. It caused to decreasing level of antibodies and increasing preventative effect on the innate immune localized at the cellular. Combination ZnONSs with dexamethasone prevents suppression of the immune system, which was observed suppression of innate immunity, in the use of dexamethasone alone [104]. Abhinanya et al. investigated the role of ZnONSs synthesized using the extract of the *Pterocarpus Marsupium* plant for biomedical applications. Protein denaturation is caused by inflammation, and ZnONSs due to albumin protein denaturation inhibition and lipid peroxidation showed anti-inflammatory activity [105]. Ali et al. studied the anti-inflammatory activity of zinc peroxide (ZnO_2_) NPs synthesized by the coprecipitation method. ZnO_2_NPs offered an anti-inflammatory role against AN4 and PA6 strains that indicated proteinase inhibition, albumin denaturation, and membrane stabilization. Percentage reduction of inflammation of ZnO2NPs was equivalent to aspirin as a standard anti-inflammatory drug at the concentration of 2000 μg/mL. The maximum inhibition of aspirin and ZnO_2_NPs were 82.1 and 81%, respectively [106].

### Drug delivery

**Fig. 11:**
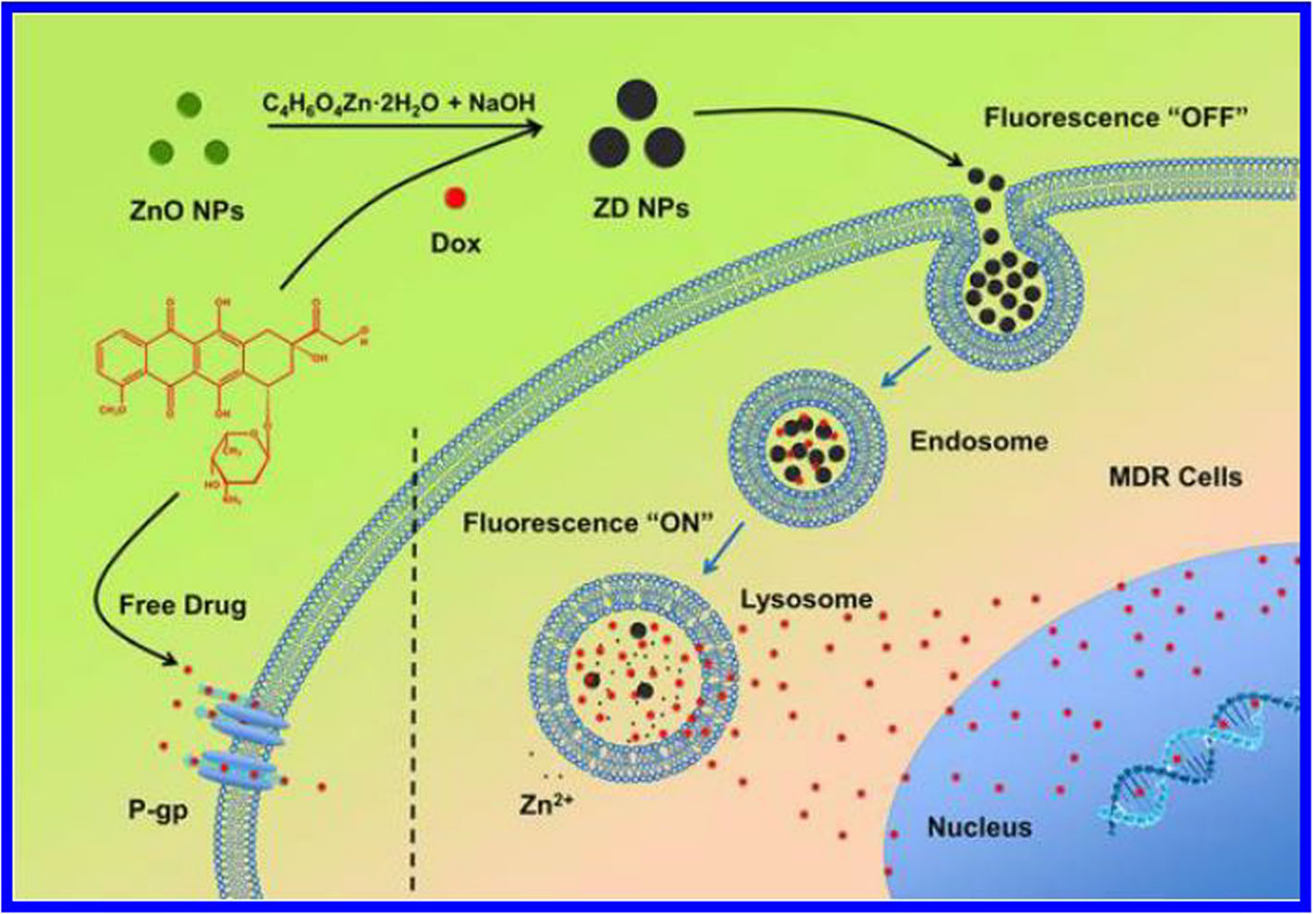
ZnONSs for drug delivery activity [109]

Due to the stable release of drugs, several nanomaterials have been used as carriers in drug delivery [107]. Shape-controlled ZnONSs are used in drug delivery due to their synergistic therapeutic effects, sustained-release profiles of the drug, long anticancer effects, and loading capacity of drugs [103, 108]. The Fig. 11 shows the role of ZnONSs in drug delivery. In this figure, the formation of ZnONSs and their effect on dominant drug resistance, displayed. Doxorubicin (DOX) is selected as a chemotherapeutic drug. Shape-controlled ZnONSs can dissolve in the tumor cells with an acidic environment, such as the lysosome and late endosome. The drug tolerated acid-triggered release of ZnONSs with the fluorescence recovery; then, it entered the nucleus to show anticancer function. The method of using ZnONSs overcame multidrug resistance [109].

Fakhar-e-Alam et al. used ZnONSs in the drug delivery approach for photodynamic therapy. In this method, by using the hydrothermal technique, PEG-ZnONSs were synthesized. Protoporphyrin IX was loaded on PEG-ZnONSs to raise the drug-carrying power capacity, and it showed superior anticancer efficiency because of drug localization in targeted targets areas. Also, mitochondrial harming efficacy led to cell necrosis due to releasing ROS [108]. For drug delivery and tissue engineering, the application of Zn loading on hydroxyapatite (HAp) with DOX bioceramic was evaluated by Kim et al. In this method, Zn loaded on HAp NPs was synthesized by the coprecipitation process. Zn doped HAp NPs showed that their properties are suitable for pH-responsive drug delivery. Doping Zinc on HAp increased bioresorbability and biocompatibility properties. Biological acting of DOX loaded on Zn and HAp (DOX-Zn-HAp) showed superior results on pH-responsive drug release for bone cancer [110]. Peng et al. synthesized core-shell structured β-CD-modified Fe3O4@ZnO: Er^3+^, Yb^3+^ nanoparticles for drug delivery. The ZnO: Er^3+^, Yb^3+^, as shell, and Fe_3_O_4_ as core acted very well for up-conversion fluorescence imaging and magnetic targeting, respectively. The ZnO: Er^3+^, Yb^3+^ shell functioned as an excellent microwave-triggered drug release to microwave radiation [111].

### Gene delivery

Gene therapy has received much attention for cancer treatment [112]. Nie et al. investigated the application of ZnONSs for plasmid DNA delivery. ZnO tetrapods could link to plasmid DNA through electrostatic interplays. The ZnO tetrapods were mixed with the cell to attach to the cell membrane. When phages were placed on cells ready for gene delivery, ZnONSs also were placed on cells ready for DNA delivery. In other words, Phages inserted genes into cells without entering the cells themselves, and ZnO tetrapods delivered DNA into cells [113]. In another study, functionalized ZnONSs were used for a polymerase chain reaction and delivery, plasmid DNA purification, and carriers for gene delivery. For plasmid DNA purification, a reversible combination of DNA occurred with ZnO tetrapods modified by amino. Also, in this gene delivery method, phages were placed on a six-legged cell, and for DNA delivery, ZnO NSs were placed on three-legged cells [114]. Zhang et al. for plasmid DNA delivery and bioimaging, application of ZnO quantum dot with poly (methacrylic acid), copolymers on poly (2- (dimethylamino) ethyl methacrylate) (ZnO QD @ PMAA-co-PDMAEMA) were investigated. QD@PDMAEMA was synthesized by a radical polymerization method. Modification on ZnO QDs caused plasmid-DNA condensation into the nanocomplexes and gene delivery [115]. Moghaddam et al. investigated apoptosis and arrest in MCF-7 as cancer cells induced by ZnO NPs. Genes such as JNK, Bax, p21, and p53 were increased and upregulated as apoptotic genes, while ERK1/2, AKT1, and Bcl-2 were decreased and downregulated anti-apoptotic genes in a dose-dependent manner [116]. Liu et al. used zinc-amine coordination for gene delivery.

**Fig. 12:**
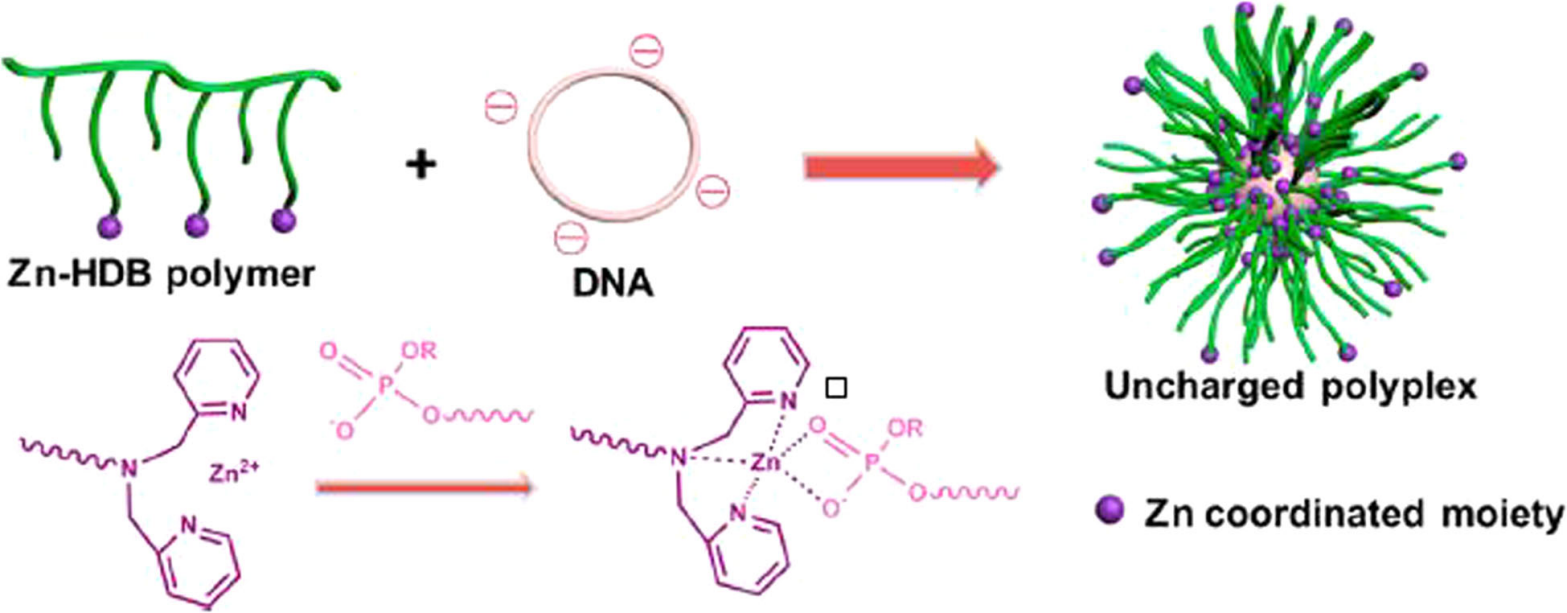
Zn-HDP polymer for condensation of DNA in a gene delivery process [117]

Coordinative zinc ligands made possible the potential of gene delivery. Coordinative zinc had an essential task in integrating gene delivery in noncationic polymers and amine substitution. Also, the simultaneous existence of hydrophobic and hydrophilic sections caused gene delivery cellular uptake and particle durability [117]. In Fig. 12, Zn coordinated shows a primary role in gene delivery. The none-cationic Zn-HDB polymers condensated on DNA and led to powerful interactions between phosphodiesters and Zinc (II)-dipicolylamine (Zn-DPA) ligand. Zn-coordinated moieties had the potential to place on the polyplex surface for endosomal disruption by interacting with phosphate bilayer [117].

### Autophagy

**Fig. 13:**
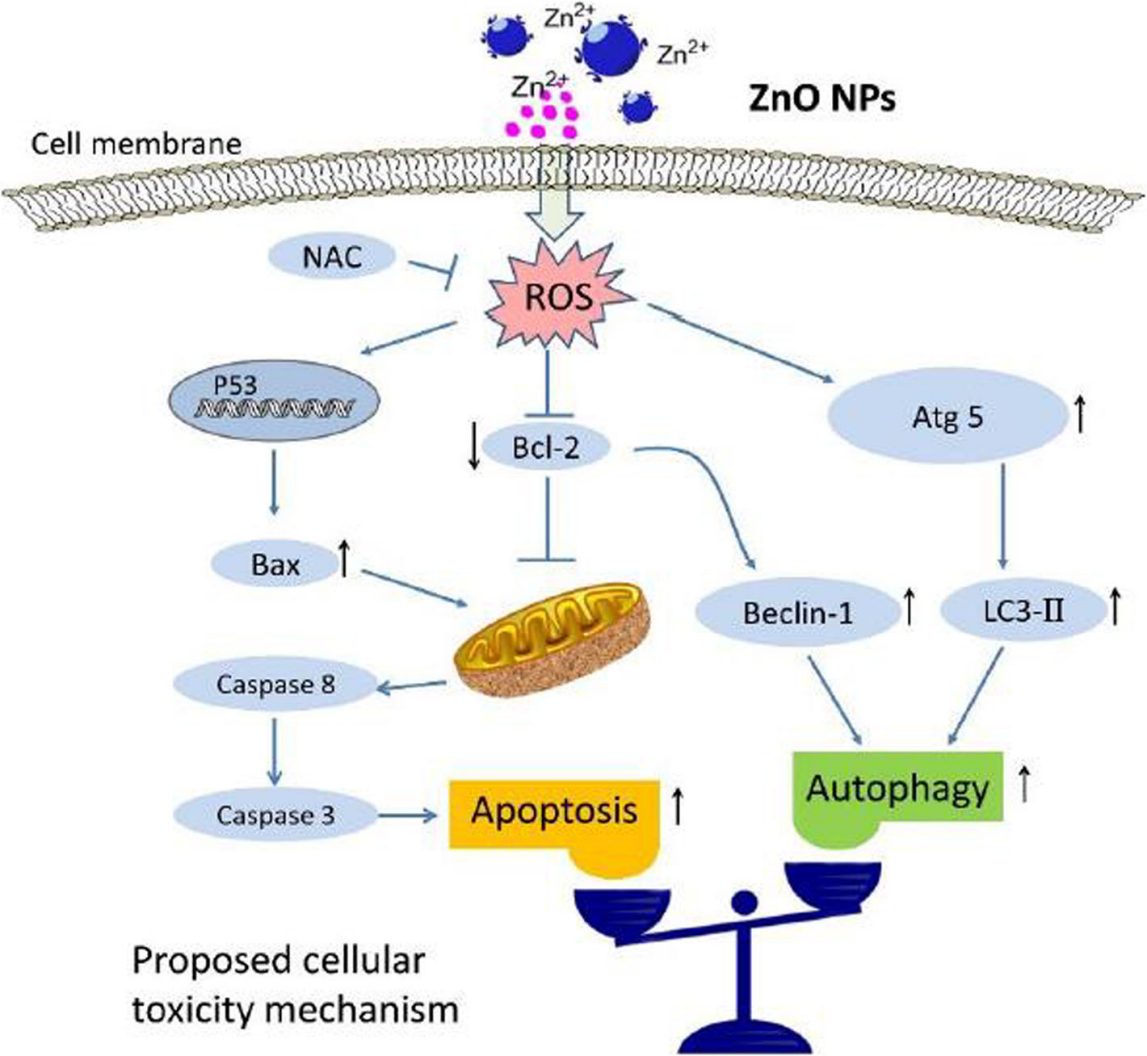
Application of Zinc in autophagy [78]

Autophagy is a main intracellular path for the destruction and recovery of long-lived proteins and damaged organs. The process of cellular detoxification and self-medication is a mechanism for cell defense. When enough food does not reach the cell or the cell has the task of killing the invader such as bacteria and viruses, autophagy helps to remove the waste and reduce the consumption of the cell and the cell, destroys the debris or the cells, and uses their raw materials to create new components. Shape-controlled ZnONSs can cause autophagy in the gastrointestinal tract, skin, immune, and kidney tissue. The role of autophagy in ZnONS was investigated by Shen et al. Results showed that the mice exposed to ZnONSs and caused to rupture of the testicular seminiferous epithelium decreased sperm density in the epididymis. There was a significant decrease in serum testosterone levels. In vitro tests demonstrated that ZnONSs produce oxidative stress, induced autophagy, and apoptosis [78]. Figure 13 shows that ZnONSs can induce autophagy and apoptosis. The apoptosis rate was measured in the presence or absence of an autophagy inhibitor after ZnONS treatment. Induction of apoptosis in TM3 mouse Leydig cells is performed by an autophagy inhibitor such as ZnONS [78].

Bai et al. investigated the role of ZnONSs inducing autophagy and apoptosis in ovarian cancers. Results showed that cells exposed to ZnONSs showed enhanced ROS production, apoptotic features such as loss of cohesion and becoming rounding, dose-dependent loss of cell viability, DNA double-strand break, upregulation of LC3 and p53, loss of mitochondrial membrane potential. Generally, ZnONSs could induce significant autophagy, apoptosis, and cytotoxicity in human ovarian cells via oxidative stress and ROS production [118].

### Tissue engineering

**Fig. 14:**
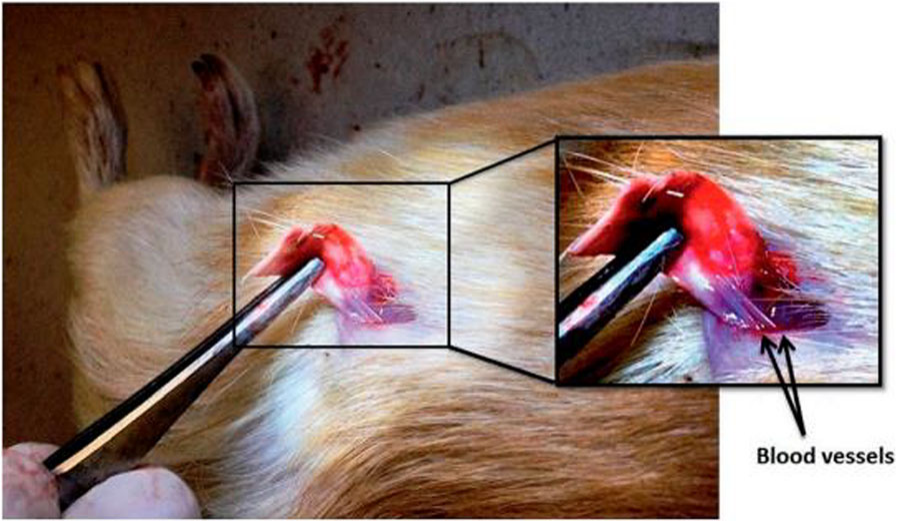
Blood vessel maturation implanted through polycaprolactone scaffolds containing 1% wt by weight of zinc oxide nanoparticles after 20 days of subcutaneous implantation [126]

Intelligent biocompatible materials have become one of the most successful approaches in the design of artificial scaffolds for tissue engineering (TE) today [119]. To support cell growth from the beginning to the end of the regeneration process and promote the formation of new tissue, nano scaffolding was used as a temporary porous structure [120]. To build new bones, organs and tissues for the different scaffolding materials subsequently implanted in the host, tissue engineering was used as a technology [121]. A strong tissue engineering scaffold repairs and regenerates damaged tissues and maintains cell attachment and cellular growth [122]. There are many limitations to the use of conventional scaffolding, such as lack of electrical conductivity, lack of adhesion, and poor mechanical strength. Scaffolding in order to increase the therapeutic potential and overcome the mentioned limitations, can be combined with nanomaterials. Researchers have recently illustrated the applications of various shape-controlled ZnONSs tissue engineering applications. Park et al. developed zinc oxide nanoflowers on a silicon substrate using the hydrothermal method and set efficient osteoblast cell growth (MC3T3-E1) to form more active filopodia and well-developed F-actin on nanoflowers compared to that on ZnO film [123]. Synthesis of highly porous electrospun PCL scaffolds containing ZnO-NPs was performed by Augustine et al. Compared to control experiments, they examined their potential in assays chicken chorioallantoic membrane, which showed excellent vascular germination (angiogenesis) and induction of increased proliferation of human cutaneous fibroblasts (HDFs) [124]. Most nanocomposite scaffolds have been implanted subcutaneously in guinea pigs by the authors. The onset of in vivo angiogenesis in the presence of nanocomposite scaffolds indicates the migration of a significant number of RBCs to those scaffolds compared to without the integration of shape-controlled ZnONSs. For tissue engineering applications with increasing integration of scaffolds and host tissue due to the anti-angiogenic properties of ZnONSs-based scaffolds with controlled shape, the authors guessed the usefulness of these scaffolds [125]. Observation of angiogenesis in doped PCL membranes in guinea pigs, after five days of subcutaneous implantation was performed In addition, a large number of red blood cells towards the membrane, along with a large numbers of fibroblasts migrated from the sides of the scaffold to the interior part [126]. Figure 14 Indicates the formation of two large blood vessels that, after 20 days of implantation, pass through the implanted subcutaneous membrane and are visible [124].

### Bioimaging

The visualizing the transformation of function and structure of biological systems, critical practice of capturing and processing is called bioimaging [100, 127]. To diagnose various diseases including cancer and cardiovascular, a non-invasive method such as bioimaging was used. Among the bioimaging techniques that are currently available are: computed tomography (CT), radiographic imaging, magnetic resonance imaging (MRI), fluorescence imaging, and positron emission tomography (PET) [128]. Recently, the evolution of several nanoparticles that known as multifaceted bioimaging tools, has taken place in a variety of pharmaceutical applications, including monitoring the delivery of biomolecules, visualizing living cells, tracking cells, and diagnosing diseases. Currently, the development of theranostic nanoparticles, which in addition to meeting dual goals with a single strategy, can be used as bioimaging and therapeutic agentsagents and scientists are also focused on developing it [129].. Shape-controlled ZnONS among different nanoparticles, due to their inherent luminescence properties and straightforward operation with fluorescence and other imaging components, became particularly interested in bioimaging applications [130]. Hong et al. Factor that targets CD105 protein at the endothelial cell surface of the tumor, such as TRC105 antibodies and 1,4,7-triacetic acid (NOTA) - conjugated ZnO-NPs and S-2- (4-isothiocyanatobenzyl) - 1,4-Triazacyclonone, which is performed by PEGylation followed by 64Cu [131] labeling, is used to form a ^64^Cu-NOTA-ZnO-PEG-TRC105 nanocomposite system [131]. ZnONS-based nanocomposite systems can act as a dual imaging agent in mice containing 4 T1 tumors for fluorescence imaging and PET imaging, as reviewed by the authors.

## Future perspectives

shape-controlled ZnONS has attracted a lot of attention from biomedical researchers due to its adjustable physicochemical properties such as size, morphology, surface charge, etc., which can be useful for their medical applications. So, the next generation of shape-controlled ZnONS can be much more effective in agents such as nano-cancer, diabetes treatment, anti-inflammatory activity, antibacterial activity, gene transfer, drug delivery, autophagy, tissue engineering and bioimaging. In addition, the appropriate morphology of shape-controlled ZnONS may increase their recognition of biological targets. A suitable strategy to change their biological effects if necessary depending on the situation, modification of physicochemical properties is shape-controlled ZnONS. However, more research relating to their stability under physiological situations, the activity of the NPs, structure, and size will be helpful and may help design anticancer nano-agents for remedial usage. Since the Shape-controlled ZnONSs showed excellent anti-inflammatory antioxidant and anti-inflammatory activities, Zinc NPs must be suggested as a high potential active agent for biomedical applications as drug delivery procedures. In this regard, scientists involved in shape-controlled ZnONS-based nanomedical projects, need to be more focused on putting their materials in clinical trials, so that the new shape-controlled ZnONS will evolve in the near future. The shape-controlled ZnONS-based biomedical research field can be expected to continue to grow in the coming decades. These nanostructures finally by the relevant authorities to be approved for the diagnosis and treatment of various diseases.

## Conclusion

This paper investigates existing strategies for controlled shape synthesis and growth processes of zinc oxide nanostructures. Also, most current techniques for the controlled synthesis of the nanostructured form of zinc oxide are obtained by changing the growth process. The growth process is the most probable path to the controlled synthesis of nanostructures. Therefore, a set of factors and principles must be used to prepare various zinc oxide nanostructures with unique properties. Based on the results, shape-controlled ZnONSs, due to activating the apoptotic signaling pathway, ROS generation, and having many inhibitory effects against bacteria and cancerous cells, have a high potential candidate as an antibacterial and anticancer agent. Therefore, it can be said that shape-controlled of ZnONSs have various applications in medicine and biology that cause them to be attractive with great practical potential. Finally, the results indicate the physicochemical properties, synthesis methods, zinc nanoparticle shape, and recent advances in shape-controlled ZnONS in the field of biomedicine. Increasing knowledge of the properties and role of shape-controlled ZnONS in biomedical therapy such as anticancer, anti-diabetes, anti-inflammatory, drug delivery, gene development, autophagy activity, tissue engineering, and bioimaging has been a significant motivation for research in this review study.

## Data Availability

All data generated or analyzed during this study are included in this published article.

## Abbreviations

ZnO: Zinc oxide
XRD: X-ray diffraction
TEG: triethylene glycol
DEG: diethylene glycol
ZnONSs: zinc oxide nanostructures
SEM: Scanning electron microscopy
TEM: transmission electron microscopy
DSSCs: dye-sensitized solar cells
NaOH: Sodium hydroxide
AFM: Atomic force microscopy
FTIR: Fourier-transform infrared spectroscopy
PEG: polyethylene Glycol
Aerosol OT: sodium bis (2-Ethylhexyl) sulfosuccinate aerosol
PTMS: propyltrimethoxysilane
BC: bacterial cellulose
ROS: reactive oxygen species
UV: ultraviolet light
PDA: polydopamine
PCE: photothermal conversion efficiency
EPR: enhanced permeability and retention
FRET: fluorescence resonance energy transfer
OGTT: oral glucose tolerance test
TSLP: lymphopoietin
iNOS: inducible nitric oxide synthase
HMG-1: high mobility group box 1
ZnO_2_: zinc peroxide
DOX: Doxorubicin
Hap: hydroxyapatite
QD: quantum dot
